# Examining Body Satisfaction and Emotional–Social Intelligence among School Children: Educational Implications

**DOI:** 10.3390/ijerph17062120

**Published:** 2020-03-23

**Authors:** Diana Amado Alonso, Benito León-del-Barco, Santiago Mendo-Lázaro, Damián Iglesias Gallego

**Affiliations:** 1 Centre for Sport Studies, Physical Education Area, Rey Juan Carlos University, Alcorcón, 28922 Madrid, Spain; 2 Department of Psychology, Teacher Training College, University of Extremadura, 10003 Cáceres, Spain; bleon@unex.es (B.L.-d.-B.); smendo@unex.es (S.M.-L.); 3 Physical Education & Exercise Lab, Teacher Training College, University of Extremadura, 10003 Cáceres, Spain

**Keywords:** body image, emotional intelligence, children, education

## Abstract

In childhood, the perception of body image is in the construction phase and emerges linked to the aesthetic ideals of society, which is well differentiated according to gender. In this way, according to people’s interpretations of the environment and how to manage it emotionally, greater or lesser body satisfaction may be generated, which could have irreversible consequences for children. Therefore, our interest lies in how body image satisfaction and gender can act as modulating variables of emotional intelligence in childhood, analyzing differences in the intrapersonal, interpersonal, stress management, adaptability, and mood dimensions of emotional intelligence, according to the degree of body image satisfaction and the child’s gender. A total of 944 Primary Education students selected by multistage cluster sampling, 548 boys and 396 girls aged between 9 and 12 years from different schools in Extremadura (Spain), participated in the research. The study design was descriptive, and questionnaires to measure emotional intelligence, self-perception, and body image satisfaction were used. An analysis of descriptive statistics, a Chi-square test to measure the variance/invariance of the participants’ distribution according to their satisfaction with body image and gender, and a MANOVA to determine the possible effects of satisfaction with body image as well as of gender on emotional intelligence were conducted. Regardless of gender, children who were satisfied with their body image showed higher interpersonal intelligence, greater adaptability, and better mood. With respect to gender, girls showed higher stress management than boys. Throughout Compulsory Education, it is necessary to promote campaigns imparted by specialists to prevent body image dissatisfaction, so that the benefits can reach the entire educational community (students, teachers, and parents). In this work, several possibilities are described to meet the demands of contemporary society.

## 1. Introduction

The term emotional intelligence was introduced by Salovey and Mayer [1] referring to the ability to understand and manage emotions to channel them in a positive way so that they work for us and not against. There are three main models of emotional intelligence: skill model or four branches of Mayer and Salovey [2], Goleman’s emotional competencies model [3,4] and Bar-On’s emotional and social intelligence model [5].

The Bar-On [5] emotional and social intelligence model is defined as an array of interrelated emotional and social competencies, skills and behaviors that determine how well we understand and express ourselves, understand others and relate with them, and cope with daily demands, challenges and pressures [6]. 

These competencies and skills have been grouped into five dimensions or factors for further study: 1) intrapersonal (the ability to understand our emotions and communicate them to others), 2) interpersonal (the ability to understand and appraise the emotions of others), 3) stress management (the ability to manage and control our emotions), 4) adaptability (flexibility and efficacy to resolve conflicts), and 5) mood (the ability to maintain a positive attitude in life) [3]. Based on these dimensions, a social-affective profile, based on a person’s emotional and social skills, which help them to operate in society daily, can be created [1,4].

Focusing on the stage of childhood as the object of this study, emotional intelligence takes on an essential role, because children’s nervous system, psyche, and personality are still in formation and construction [5,6,7,8]. Hence, many of the feelings, emotions, and perceptions that arise at this stage can become consolidated as beliefs, contributing to generate a series of consequences that may emerge during later stages and that can condition people’s personality and their lifestyle [9,10,11].

In this regard, one of the variables that is receiving increasing attention in developmental stages like childhood and adolescence is body image [12,13,14], as it is being formed according to the individual’s subjective ratings of his or her own body, and to the social appraisals received from others [15]. In this sense, the way people perceive, feel, and behave in relation to their own body may be associated with high body satisfaction or body dissatisfaction. If not properly controlled or directed, this can cause serious consequences, such as the development of eating behavior disorders [16,17].

In this line, the scientific literature has highlighted the link between body satisfaction/dissatisfaction and emotional intelligence, based on the fact that cognitive processes are directly affected by the management of emotions [18,19]. In fact, people’s adaptive and accurate beliefs or perceptions are often associated with adequate management of emotions and, therefore, greater emotional intelligence, contrary to what is observed in maladaptive and inaccurate beliefs [20].

However, body satisfaction is determined by the aesthetic ideal prevailing in society, which currently focuses on the cult of the body, youth, and beauty as a goal in order to achieve social success [21]. Therefore, when we speak in terms of the canons of beauty in society, it is imperative to differentiate according to gender, as aesthetic ideals differ in men and women. In our Western society, men seek a body image where their muscles predominate, whereas women pursue thinness [22,23].

Thus, taking into account the role of body satisfaction/dissatisfaction and gender in emotional intelligence, the main objective of this work is to provide information and/or deepen our knowledge about the modulating variables of emotional intelligence in childhood. For this purpose, we intend to analyze the differences in the dimensions of emotional intelligence depending on children’s degree of satisfaction with their body image and their gender. These factors and their joint study are taken into consideration, given the interest and relevance of the study of body image at these ages, with gender being a possible determinant in relation to emotional intelligence and body satisfaction. 

The main aim of this study is to know how body image satisfaction and gender can act as modulating variables of emotional intelligence in childhood, analyzing differences in the intrapersonal, interpersonal, stress management, adaptability, and mood dimensions of emotional intelligence, according to the degree of body image satisfaction and children’s gender. Based on this aim, the hypothesis proposed is that participants who show greater satisfaction with their body image will have higher levels of emotional intelligence. 

## 2. Materials and Methods 

### 2.1. Participants

Participants in the investigation were 944 students from 5th and 6th grade of Primary Education, 548 boys (58%) and 396 girls (42%), aged between 9 and 12 years (M = 10.76, SD = 1.11). The participants were from eight schools of the Autonomous Community of Extremadura (Spain). Sample selection was done by multistage cluster sampling, randomly selecting the groups in the schools that had various subgroups in the above-mentioned grades of Primary Education.

### 2.2. Instruments

#### 2.2.1. Emotional Intelligence

To measure children’s emotional intelligence in the primary education stage, we used the Emotional Quotient Inventory: Young Version (EQ-i: YV), designed by Bar-On and Parker [4] and validated in Spanish by Ferrándiz, Hernández, Bermejo, Ferrando and Sáinz [3]. This instrument consists of 60 items that make up the global factor Emotional Intelligence, divided, in turn, into five dimensions: Intrapersonal (6 items: emotional self-awareness, assertiveness, personal respect, self-actualization, independence), Interpersonal (12 items: interpersonal relationships, social responsibility, empathy), Stress Management (12 items: stress tolerance, impulse control), Adaptability (10 items: problem solving, reality appraisal, flexibility), and General Mood (14 items: joy, optimism). To these 60 items were added another 6 items that make up a Positive Impression Scaleto measure, the degree to which subjects respond randomly or distort their responses as a function of social desirability. The answers to the questionnaire were rated on a four-point Likert scale ranging from 1 (never happens to me) to 4 (always happens to me). 

The EQ-i: YV presented a structural model with good adjustment rates (χ^2^ = 24,780, df = 5, *p* < 0.001; NFI = 0.966; CFI = 0.973; IFI = 0.973; RMSEA = 0.069) and had adequate internal consistency for the total number of items that constitute the global Emotional Intelligence factor, with a Cronbach alpha coefficient of 0.84. The Cronbach alpha indexes (α = 0.84) and composite reliability (CR = 0.80) indicate an adequate overall reliability of the EQ-i: YV with an average variance extracted (AVE) of 0.50. Likewise, the dimensions or factors of the questionnaire present an acceptable reliability and AVE ≥ 0.50 [Intrapersonal (α = 0.67, CR = 0.82, AVE = 0.50); Interpersonal (α = 0.70, CR = 0.90, AVE = 0.56); Stress management (α = 0.69, CR = 0.87, AVE = 0.56); Adaptability (α = 0.73, CR = 0.88, AVE = 0.54); and Mood (α = 0.72, CR = 0.91, AVE = 0.57)]. Lastly, it should be noted that Items: 6, 15, 21, 26, 28, 33, 37, 46, 49, 53, 54, and 58 were worded negatively, so their scores were reversed to facilitate the process of data analysis. 

Then, the invariance of the EQ-i: YV was examined according to gender and the degree of conformity with the figure. The values found in the indexes for the Unconstrained Models, with differences less than 0.01 of the CFI indices between the four models, indicate that the factorial loads of the questionnaire are equivalent in both cases (Table 1).

#### 2.2.2. Self-Perception of Body Image

To evaluate the children’s own body image and the ideal body image, we used the Stunkard Figure Rating Scale [24]. This scale consists of 9 figures that progressively increase in size, going from very thin (value 1) to very obese (value 9). The own body image is the number of the figure selected by the participants in response to the heading “choose the figure that reflects how you think you look”. The ideal body image is the number of the figure chosen in response to the header “choose your ideal figure”. The scale has adequate validity and test-retest reliability [25]. In previous recent studies in the Spanish context and with samples of the same age as our research, the instrument was valid and reliable [26]. To be able to define one’s own body image and the ideal body image, following other authors, four categories were created to group the participants: Low body weight (values 1 and 2), normal weight (values 3 and 4), overweight (values 5, 6, and 7), and obesity (values 8 and 9).

#### 2.2.3. Satisfaction with Body Image

This variable is defined as the difference between one’s own perceived body image and the perceived ideal body image [27]. Based on this, a score of satisfaction with body image was created for each participant by subtracting the number of the figure indicated as the ideal perceived body image from the number of the figure selected as one’s own perceived body image. To facilitate the grouping of the participants and the subsequent analyses, three categories of body image satisfaction were created: satisfied (own image = ideal), dissatisfied (own − ideal = ± 1), very dissatisfied (own − ideal ≥ ± 2).

### 2.3. Procedure

This study was approved by the Bioethics and Biosafety Committee of the University of Extremadura (N. 0063/2018). All participants were treated in agreement with the ethical guidelines of the American Psychological Association with respect to consent, confidentiality, and anonymity of answer.

We followed a protocol to ensure that data collection was carried out in a similar manner throughout the process. First, we contacted each of the schools and requested a meeting with the director for any questions about aims, goals, time, and grades that could be involved in the research. After they accepted the proposal, the director set up a meeting with the teachers, and we proposed a suitable schedule for the administration of the tests. In addition, all participants received an informed consent form to be signed by their parents or legal guardians, authorizing their participation in this research, as they were aged between 9 and 12 years. 

Prior to the collective administration of the questionnaires during the agreed schedules, the main investigator briefly explained the procedure, as well as the instructions to complete the questionnaires, informing the participants that the data they provided would be anonymous and confidential. The duration was approximately 30 to 40 minutes for each class, and the main investigator was present at all times to clarify any doubts that could arise during the process. 

At the end of data collection in each class, a brief informal interview was held with the teachers to discuss some aspects or extraneous variables to be taken into account in some students. Likewise, after completing the process in each school, we met the directors to thank them and discuss the process, ensuring the availability of the research data at any time.

### 2.4. Data Analysis

The factorial structure of the EQ-I: YV was evaluated using the Cronbach alpha coefficient, the CR, and the AVE. The Chi-square test establishes the variance/invariance of the participants’ distribution according to their satisfaction with body image and gender. The data was submitted to the Kolmogorov–Smirnov test to analyze the assumption of normality, the Rachas test to contrast the assumption of randomization, and the Levene test to contrast the assumption of homocedasticity. We found that *p* > 0.05 in all contrasts, so the use of parametric tests was justified. In order to determine the possible effects of satisfaction with body image as well as of gender on emotional intelligence, a multivariate analysis of variance (MANOVA) was conducted, in which the dependent variables corresponded to the five dimensions of Emotional Intelligence, and the independent variables were children’s satisfaction with body image and gender. The assumptions of normality, randomization, and homoscedasticity were contrasted with the Kolmogorov–Smirnov, Rachas, and Levene tests, respectively. 

## 3. Results

Table 2 presents the distribution of the participants according to their body image satisfaction and gender.

As for body image satisfaction, it should be noted that 76.3% of the participants were not satisfied with their body image, specifically 55% preferred to be thinner and 20% preferred to be heavier/more athletic. In relation to gender, the distribution of the groups’ satisfaction with body image was equivalent (χ2(2) = 2.237, *p* = 0.327). However, there were differences (χ2(2) = 8.471, *p* = 0.014) in relation to the feeling of dissatisfaction because, although the percentage of girls (56%) and boys (54.3%) who preferred to have a thinner build was equivalent (*p* > 0.05), the percentage of boys (23.2%) who preferred to have a more athletic build was higher (*p* ≤ 0.05) than that of the girls (15%).

Table 3 shows the means and standard deviations obtained for Emotional Intelligence as a function of the groups’ satisfaction with body image and gender.

To analyze the effect of body image satisfaction and gender on emotional intelligence, a MANOVA was carried out, including as independent variables body image satisfaction (satisfied, dissatisfied, and very dissatisfied) and gender (female and male) and, as dependent variables, all the dimensions of emotional intelligence (intrapersonal, interpersonal, stress management, adaptability, and mood).

The MANOVA found significant main multivariate effects of body image satisfaction (Wilks λ = 0.881, F(10, 1640) = 10.678, *p* < 0.001, ƞ = 0.061), gender (Wilks λ = 0.968, F(5, 820) = 5.503, *p* < 0.001, ƞ = 0.032), and their interaction (Wilks λ = 0.978, F(10, 1640) = 1.869, *p* = 0.045, ƞ = 0.011).

In contrast, the univariate comparisons showed the existence of a significant main effect of the degree of body image satisfaction in the emotional intelligence interpersonal components, F(2, 834) = 9.026, *p* < 0.001, η = 0.021, stress management, F(2, 834) = 3.348, *p* = 0.036, η = 0.008, adaptability, F(2, 834) = 9.612, *p* < 0.001, η = 0.023, and mood, F(2, 834) = 51.23, *p* < 0.001, η = 0.111, with no significant main effect in the intrapersonal component, F(2, 834) = 2.863, *p* = 0.058, η = 0.007.

With regard to gender, there was a significant main effect in the emotional intelligence interpersonal components, F(1, 834) = 6.257, *p* = 0.013, η = 0.008, and stress management, F(1, 834) = 7.419, *p* = 0.007, η = 0.009, with no significant main effect in the intrapersonal components, F(1, 834) = 2.977, *p* = 0.085, η = 0.004, adaptability, F(1, 834) = 3.267, p = 0.071, η = 0.004, or mood, F(1, 834) = 0.778, *p* = 0.378, η = 0.001. In addition, a significant interaction was found between body image satisfaction and gender in the stress management component, F(2 834) = 3.636, *p* = 0.027, η = 0.009.

The multiple comparisons of body image satisfaction, gender, and their interaction indicate that children who are satisfied with their body image obtained higher scores (p ≤ 0.05) than people who were very dissatisfied in the interpersonal, adaptability, stress management, and mood components of emotional intelligence, although in the component stress management, the differences between satisfied and very dissatisfied individuals were only significant in boys, with no differences (*p* ≤ 0.05) in stress management as a function of body image satisfaction among girls. On the other hand, the subsequent inter-subject analysis of gender effects revealed significant differences in interpersonal components and stress management, such that girls scored higher (*p* ≤ 0.05) than boys in all the paired comparisons of body image satisfaction (satisfied, dissatisfied, or very dissatisfied), except for the comparison between boys and girls who were satisfied with their body image in the interpersonal component, where no differences were found (*p* ≤ 0.05) between boys and girls.

## 4. Discussion

The main objective of this study was to analyze the differences in the intrapersonal, interpersonal, stress management, adaptability, and mood dimensions of emotional intelligence in childhood as a function of the children’s degree of body image satisfaction and gender. Based on this aim, the hypothesis proposed is that participants who show greater satisfaction with their body image will have higher levels of emotional intelligence.

As a starting point and a relevant general fact, the results show that 76% of the children are not satisfied with their body image: the girls want to be thinner and the boys prefer to have a more athletic build. These results could be explained partly by the age of the sample, as a preadolescent stage where children are starting to mature, with all the changes that this entails in their body. In fact there are more and more studies that show neurobiological and social contextual factors that could influence the development of social cognition and behavior during adolescence and puberty [28]. However, if we focus on today’s society this indicates that children rate their body negatively, influenced by the aesthetic ideal implanted in contemporary society [21], which rewards thinness in the female body and musculature in the male body, moreover, seeking beauty and absolute perfection [22,23].

These data are alarming, taking into account the studies that have shown that body dissatisfaction is linked to negative and maladaptive consequences of children’s behavior, such as the development of eating behavior disorders [16,17], and low levels of physical activity [29]. Therefore, before such dissatisfaction can lead to behaviors that are consolidated and harmful for children’s health, it is necessary to intervene in the schools, where the subject of Physical Education could be a crucial aspect [30,31].

However, if we focus on the age of the sample, as a preadolescent stage, we must consider that children are starting to mature, with all the changes that this entails in their body. Focusing on the hypothesis of the present study, the results show that, regardless of gender, children who have better emotional and social competencies, greater adaptability, and better mood are also more satisfied with their body image, so the hypothesis can be confirmed. That is, children who are satisfied with their body image have greater skills to understand and appreciate the emotions of others, greater flexibility and effectiveness to resolve conflicts, and a more positive life attitude, and therefore, they can better control their emotions. In line with these findings, it has been shown that emotions, perceptions, and thoughts are interconnected [18,19], so if we learn to control our emotions and extract relevant information, this affects the creation of more accurate, precise, and positive perceptions and thoughts [20]. 

However, the results indicate that, regardless of the level of satisfaction with body image, girls show higher stress management than boys. These findings seem to be influenced by age because, in adolescence, girls show higher levels of stress perception [32,33] so, considering the stage in which the present study is contextualized (encompassing childhood and preadolescence) as well as girls’ early maturation compared to boys, girls’ greater perception of stress may be related to the physiological, psychological, and social changes they are experiencing [34]. These gender differences in the perception of stress have been explained by biology and by the expectations of the society in which girls are born [35], where women are expected to present great skills within and outside of the educational context and to tend toward self-perfection [36]. These demands have contributed to women’s learning to self-manage in order to cope with everything and improve their individual performance each day, so that they are more likely to manage their stress better and to present higher self-esteem due to their performance in all their settings [33].

## 5. Limitations

One of the main limitations of the study is that we did not really measure the students’ size or weight. This prevents knowing whether their body image satisfaction/dissatisfaction was only a perception provoked by the need to meet the aesthetic ideals implanted in the present society or it was a realistic perception, where their weight is far from the average weight established for people of their age and gender. Therefore, it would be necessary to complement the measure of body image satisfaction/dissatisfaction with the real measure of the children’s size and weight to determine whether or not they have health problems which should be resolved more urgently and from other approaches.

On the other hand, it should be noted that a research sample is a possible limitation in terms of reliability of information, because it cannot be generalized to other types of populations or children with different ages.

## 6. Conclusions

The following main conclusions of this work can be reached: (1) 76.3% of the children participating in this research are not satisfied with their figure, the boys want to have a more athletic build; (2) children who are satisfied with their body image show more emotional skills to relate with others, greater adaptability to any situation, and better mood; (3) girls have a greater ability to manage stress and have more emotional control than boys. 

With a view to subsequent studies, as primary and secondary education is compulsory and can reach all the children and young people, it would be necessary to promote training and intervention programs to prevent body image dissatisfaction, imparted by psychologists, graduates in sports sciences, nutritionists, and even doctors. The objective would be to raise the awareness of the entire educational community (students, teachers, and parents) and to implement practical strategies to maintain the body that we desire in a healthy way, through eating habits (by offering healthy menus at school) and the practice of physical activity. 

In order to awaken interest in these kinds of programs and make them effective, they must be adapted to the demands of contemporary society. Therefore, the use of new technologies could be applied, such as the creation of educational blogs in which students participate actively or the use of mobile applications for adequate consumption of food and for the practice of physical activity [37,38]. It is also interesting for participants to experience all this in workshops designed by specialists in each of the affected areas, so that prevention is reinforced with active, cooperative, and collaborative learning.

The main contribution of this study to the scientific literature is that most of the studies carried out have observed the influence of emotional intelligence on body satisfaction (usually linked to eating disorders). However, in this study we observed how body satisfaction and gender can modulate emotional intelligence, specifically studying psychological variables, the way people perceive, feel, and behave in relation to their own body. Thereby, we could promote training and intervention programs at schools to promote good emotional and social skills in children.

## Figures and Tables

**Table 1:** Multigroup analysis of invariance by gender and degree of conformity with the figure.

Groups	Models	χ2	df	χ2/df	Δχ2	*p*	Δdf	CFI	TLI	RMSEA
Gender	Unconstrained	38.319	10	3.812	-		-	0.962	0.925	0.058
	Measurement weights	43.906	14	3.136	5.588	0.232	4	0.960	0.943	0.051
	Structural covariances	44.732	15	2.982	6.413	0.268	5	0.960	0.947	0.049
	Saturated model	58.284	20	2.914	19.965	0.030	10	0.949	0.949	0.048
Body Image Satisfaction	Unconstrained	58.829	15	3.922	-		-	0.940	0.920	0.059
	Measurement weights	93.090	23	4.047	34.261	<0.001	8	0.904	0.916	0.061
	Structural covariances	95.775	25	3.831	36.946	<0.001	10	0.903	0.918	0.059
	Saturated model	132.686	35	3.791	75.857	<0.001	20	0.902	0.910	0.058

**Table 2:** Distribution of Participants according to Body Image Satisfaction and Gender.

Body Image Satisfaction	Female		Male		Total	
	*n*	*%*	*n*	*%*	*n*	*%*
Satisfied	104	26.3	120	21.9	224	23.7
Dissatisfied	214	54	324	59.1	538	57
Very dissatisfied	78	19.7	104	19	182	19.3
Total	386	100	534	100	944	100

**Table 3:** Means and Standard Deviation of Emotional Intelligence Dimensions according to Body Image Satisfaction and Gender.

EI Dimensions		Female		Male		Total	
		*M*	*SD*	*M*	*SD*	*M*	*SD*
Intrapersonal	Satisfied	14.92	2.71	14.92	3.50	14.92	3.16
	Dissatisfied	14.56	3.58	14.02	3.35	14.24	3.45
	Very dissatisfied	14.85	3.60	13.70	2.79	14.11	3.13
	Total	14.69	3.33	14.22	3.36	14.41	3.36
Interpersonal	Satisfied	42.76	5.50	42.60	5.02	42.67	5.23
	Dissatisfied	41.91	5.06	40.09	5.19	40.81	5.21
	Very dissatisfied	41.46	2.02	39.61	7.37	40.28	6.06
	Total	42.14	5.04	40.67	5.49	41.27	5.36
Stress management	Satisfied	33.45	5.43	32.81	6.99	33.10	6.33
	Dissatisfied	32.95	5.29	31.49	5.55	32.47	5.44
	Very dissatisfied	33.08	5.68	28.63	5.21	30.56	5.67
	Total	33.14	5.36	32.26	5.99	32.48	5.75
Adaptability	Satisfied	28.86	4.54	30.60	5.15	29.81	4.95
	Dissatisfied	27.98	4.73	28.78	4.86	28.46	4.82
	Very dissatisfied	27.15	4.27	27.17	4.76	27.17	4.56
	Total	28.18	4.65	29.09	5.01	28.72	4.89
Mood	Satisfied	49.25	5.50	50.50	3.71	49.94	4.63
	Dissatisfied	47.05	5.65	46.43	5.77	46.68	5.73
	Very dissatisfied	42.31	5.10	43.09	6.32	42.81	5.89
	Total	47.35	5.82	47.15	5.80	47.23	5.80

*Note*: EI (Emotional Intelligence).

## References

[1] Salovey P., Mayer J.D. (1990). Emotional intelligence. Imagin. Cogn. Personal..

[2] Mayer J.D., Salovey P., Salovey P., Sluyter D. (1997). What is emotional intelligence?. Emotional Development and Emotional Intelligence: Implications for Educators.

[3] Goleman D. (1995). Emotional Intelligence.

[4] Goleman D. (1998). La Práctica de la Inteligencia Emocional.

[5] Bar-On R., Bar-On R., Parker J.D.A. (2000). Emotional and social intelligence: Insights from the Emotional Quotient Inventory (EQi). Handbook of Emotional Intelligence: Theory, Development, Assessment and Application at Home, School and in the Workplace.

[6] Bar-On R. (1997). The Bar-On Emotional Quotient Inventory (EQ-i): A Test of Emotional Intelligence.

[7] Bar-On R. (2006). The Bar-On model of emotional-social intelligence (ESI) 1. Psicothema.

[8] Ferrándiz C., Hernández D., Bermejo R., Ferrando M., Sáinz M. (2012). Emotional and social intelligence in childhood and adolescence: Spanish validation of an instrument for its measurement. Rev. Psicodidáctica..

[9] Bar-On R., Parker J.D. (2000). Bar-On emotional quotient inventory: Youth version. Technical manual.

[10] Briley D.A., Tucker-Drob E.M. (2014). Genetic and environmental continuity in personality development: A meta-analysis. Psychol. Bull..

[11] Cecchini J.A., Méndez-Giménez A., García C. (2018). Validation of the Emotional Intelligence Questionnaire in Physical Education. Rev. Psicol. Dep..

[12] Cicchetti D. (2016). Socioemotional, personality, and biological development: Illustrations from a multilevel developmental psychopathology perspective on child maltreatment. Annu. Rev. Psychol..

[13] Villanueva L., Prado-Gascó V., González R., Montoya I. (2014). Emotional awareness, mood and individual and social adjustment outcomes in children aged 8–12 years old. Ann. Psychol..

[14] Carr A. (2015). The Handbook of Child and Adolescent Clinical Psychology: A Contextual Approach.

[15] Cobos-Sánchez L., Flujas-Contreras J.M., Gómez-Becerra I. (2017). The role of emotional intelligence in psychological adjustment among adolescents. Ann. Psychol..

[16] Diaz-Castela M., Hale III W.W., Muela J.A., Espinosa-Fernández L., Klimstra T., Garcia-Lopez L.J. (2013). The measurement of perceived emotional intelligence for Spanish adolescents with social anxiety disorder symptoms. Ann. Psychol..

[17] Mendo-Lázaro S., Polo-del-Río M.I., Amado-Alonso D., Iglesias-Gallego D., León-del-Barco B. (2017). Self-concept in childhood: The role of body image and sport practice. Front. Psychol..

[18] Hernández-Cortés L.M., Londoño Pérez C. (2013). Body image, CMI, coping, depression and BED risk in young univesitaries. Ann. Psychol..

[19] Tatangelo G., McCabe M., Mellor D., Mealey A. (2016). A systematic review of body dissatisfaction and sociocultural messages related to the body among preschool children. Body Image.

[20] Fernandes H.M. (2018). Physical activity and mental health in adolescents: The mediating effect of self-esteem and body satisfaction. Rev. Psicol. Dep..

[21] Marco J.H., Perpiñá C., Botella C. (2014). The treatment of the body image disturbances in eating disorders and clinically significant change. Ann. Psychol..

[22] Wong Y., Lin J.S., Chang Y.J. (2014). Body satisfaction, emotional intelligence, and the development of disturbed eating: A survey of Taiwanese students. Asia Pac. J. Clin. Nutr..

[23] Hollander E., Cohen L.J., Simeon D. (1993). Body dysmorphic disorder. Psychiatr. Ann..

[24] Boden M.T., Berenbaum H. (2010). The bidirectional relations between affect and belief. Rev. Gen. Psychol..

[25] Boden M.T., Gala S., Berenbaum H. (2013). Emotional awareness, gender, and peculiar body-related beliefs. Cog. Emot..

[26] Berenbaum H., Boden M.T., Baker J.P. (2009). Emotional salience, emotional awareness, peculiar beliefs, and magical thinking. Emotion.

[27] Uhlmann L.R., Donovan C.L.Ç., Zimmer-Gembeck M.J., Bell H.S.C., Ramme R.A. (2018). The fit beauty ideal: A healthy alternative to thinness or a wolf in sheep’s clothing?. Body Image.

[28] Lampis J., Cataudella S., Busonera A., De Simone S., Tommasi M. (2019). The moderating effect of gender role on the relationships between gender and attitudes about body and eating in a sample of Italian adolescents. Eat Weight Disord..

[29] Rice K., Prichard I., Tiggemann M., Slater A. (2016). Exposure to Barbie: Effects on thin-ideal internalisation, body esteem, and body dissatisfaction among young girls. Body Image.

[30] Stunkard A.J., Sørensen T., Schulsinger F., Kety S. (1983). Use of the Danish Adoption Register for the study of obesity and thinness. the Genetics of Neurological and Psychiatric Disorders.

[31] Thompson J.K., Altabe M.N. (1991). Psychometric qualities of the Figure Rating Scale. Int. J. Eat. Disord..

[32] Sánchez G.F.L., Suárez A.D., Smith L. (2018). Análisis de imagen corporal y obesidad mediante las siluetas de Stunkard en niños y adolescentes españoles de 3 a 18 años. Ann. Psychol..

[33] Lynch E., Liu K., Wei G.S., Spring B., Kiefe C., Greenland P. (2009). The relation between body size perception and change in body mass index over 13 years the Coronary Artery Risk Development in Young Adults (CARDIA) Study. Am. J. Epidemiol..

[34] Bosacki S. (2019). Social Consequences of Pubertal Timing. The Encyclopedia of Child and Adolescent Development.

[35] Añez E., Fornieles-Deu A., Fauquet-Ars J., López-Guimerà G., Puntí-Vidal J., Sánchez-Carracedo D. (2018). Body image dissatisfaction, physical activity and screen-time in Spanish adolescents. J. Health Psychol..

[36] Ishak S.I.Z.S., Chin Y.S., Taib M.N.M., Shariff Z.M. (2016). School-based intervention to prevent overweight and disordered eating in secondary school Malaysian adolescents: A study protocol. BMC Public Health.

[37] Amado-Alonso D., Mendo-Lázaro S., León-del-Barco B., Mirabel-Alviz M., Iglesias-Gallego D. (2018). Multidimensional self-concept in elementary education: Sport practice and gender. Sustainability.

[38] MacLean A., Sweeting H., Egan M., Der G., Adamson J., Hunt K. (2013). How robust is the evidence of an emerging or increasing female excess in physical morbidity between infancia and adolescence? Results of a systematic literature review and meta-analyses. Soc. Sci. Med..

